# Platelets isolated from an Alzheimer mouse damage healthy cortical vessels and cause inflammation in an organotypic *ex vivo* brain slice model

**DOI:** 10.1038/s41598-018-33768-2

**Published:** 2018-10-19

**Authors:** Kathrin M. Kniewallner, Bettina M. Foidl, Christian Humpel

**Affiliations:** Laboratory of Psychiatry and Exp. Alzheimer’s Research, Department of Psychiatry I, Medical University of Innsbruck, Innsbruck, Austria

## Abstract

Platelets are anuclear blood cells and play a major role in hemostasis and thrombosis. Platelets express amyloid-precursor protein (APP), release beta-amyloid (Aβ) and are stimulated (pre-activated) in Alzheimer’s disease (AD). We hypothesize that such stimulated platelets severely damage brain vessels which subsequently leads to cerebrovascular damage in AD. In order to study this issue we isolated platelets from AD mice (expressing APP with the Swedish-Dutch-Iowa mutations), labeled them with the red fluorescent dye PKH26 and transcardially infused these freshly isolated platelets into the brains of anesthetized healthy C57BL6 wildtype mice. Brains were immediately taken, 110 µm thick organotypic brain slices prepared and cultured for 1 or 14 days. We observed that red PKH26^+^ fluorescent platelets were localized in collagen IV and Lectin-649 counterstained cortical brain vessels and that platelets from AD mice severely damaged cortical brain vessels in wildtype mice and entered the brain parenchyma. Confocal microscopy showed immunoreactivity for matrix metalloproteinases (MMP-2 and MMP-9) and beta-amyloid around these platelets. The effect was completely inhibited with an MMP inhibitor. Furthermore, isolated AD platelets caused inflammation and activated microglia around the site where platelets damaged cortical brain vessels. We conclude that AD-derived platelets more aggressively damage healthy vessels which may consequently play a role in the progression of cerebral amyloid angiopathy in AD.

## Introduction

Alzheimer Disease (AD) is the most common neurodegenerative disorder of the brain and is characterized by neurotoxic beta-amyloid (Aβ) plaque deposition, intraneuronal tau pathology, cholinergic neurodegeneration, inflammation and oxidative stress. These pathologies cause cognitive impairment and memory deficits. It is hypothesized that AD is a vascular disease and linked to stroke, atherosclerosis or hypertension and vascular risk factors may increase the risk for sporadic AD^1–5^. In addition, Aβ deposits are found in brain vessels, called cerebral amyloid angiopathy (CAA)^6^. CAA is one of the most frequent causes of intracerebral hemorrhage leading to vascular fragility due to degeneration of the vessel wall, formation of microaneurysm especially in cortical blood vessels^7,8^. Vascular alterations such as an increased number of fragmented vessels, altered vessel diameters and disrupted vessels are very frequent in AD^9^. However, so far it is not clear when and how Aβ is deposited in the vessel walls. Is CAA initially caused from peripherally released Aβ resulting in deposition in the vessel wall, or is CAA just a result of the high Aβ overload in the brain?

It has been hypothesized that AD is a vascular disease several years ago^3–5^ and that it begins as a disease of small blood vessels, damaged by oxidative-induced inflammation and dysregulated amyloid metabolism^10^. Indeed, such an AD vessel pathology including microbleedings is well characterized by MRI imaging^11,12^. Further, there is evidence that vessels are damaged and disrupted and that small bleedings occur during the AD pathology^13^. These bleedings may cause influx of substances from the blood into the brain, such as thrombin or IgGs, but also activate hemostasis and thrombosis. Indeed, platelets become stimulated (pre-activated) in AD^14^ and play a role in vessel repair and clotting^15,16^. In fact, platelets are very interesting blood cells in AD, because they express high amounts of amyloid-precursor protein (APP) and release Aβ (mainly the Aβ_40_ form)^17^. Although the role of peripheral Aβ is not fully clear, it may be used as a clotting substance during vessel repair^16^. However, there is strong evidence that platelets are affected during the AD progression e.g. showing increased platelet activation in AD patients, altered platelet volume, but also differential expression of biomarkers^18^. Interestingly, the ratio of the two APP isoforms is markedly altered in AD platelets^18–22^. We recently demonstrated that Thiazine Red positive platelets are early signs during the AD pathology in transgenic AD mice^23^ possibly damaging brain vessels at an early stage of AD.

**Figure 1:**
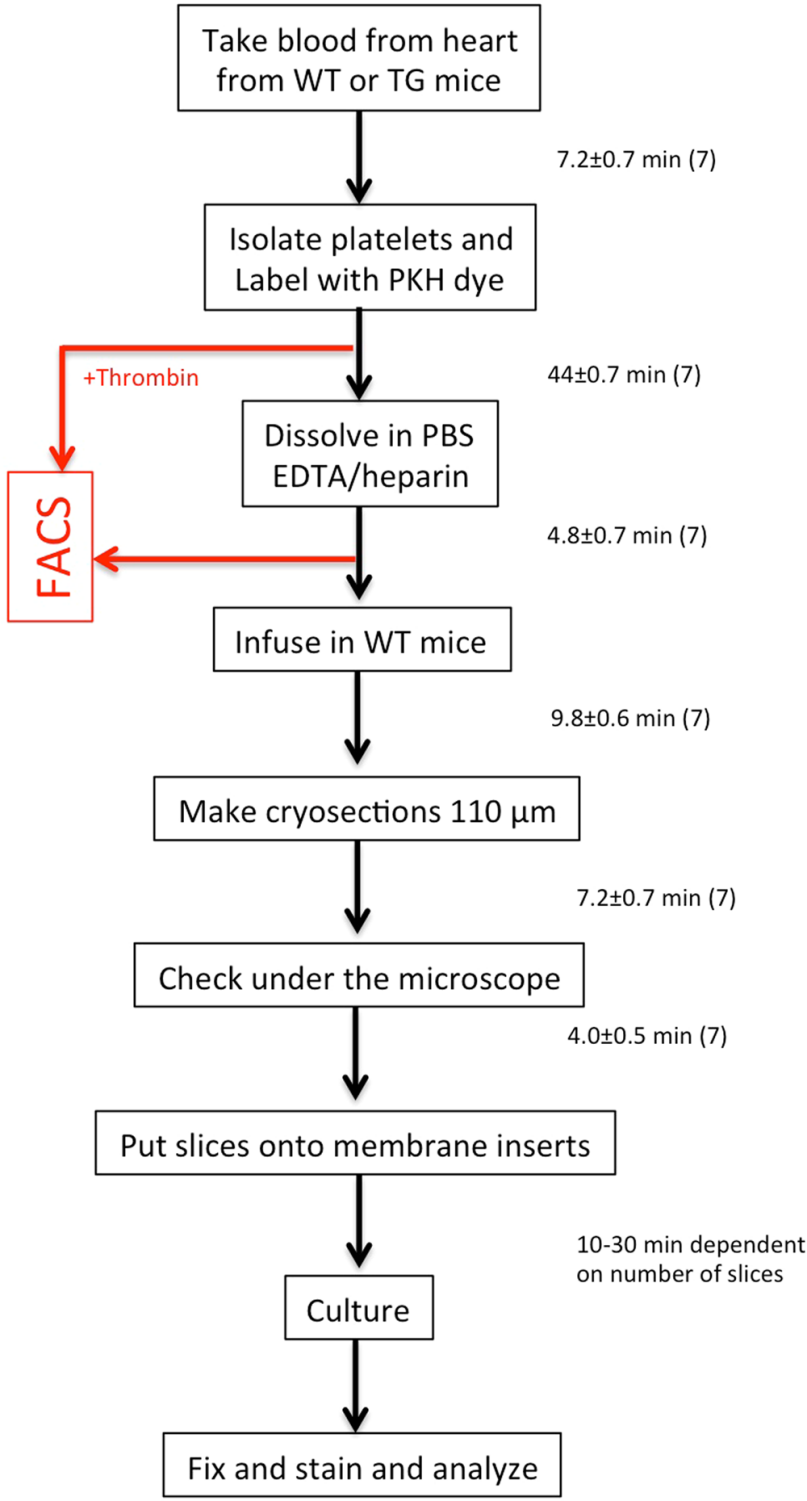
The workflow shows the experimental design and the time to perform the experiments (in mean ± SEM minutes). WT, wildtype; TG, transgenic Alzheimer mice.

In the present study, we hypothesize that platelets isolated from AD mice damage healthy brain vessels and cause CAA^24^. In order to study this issue (see Workflow Fig. 1) we will isolate platelets from wildtype (C57BL6) and transgenic AD (APP_SweDI) mice, label them with PKH26 (a red fluorescent dye) and transcardially infuse these platelets into the brains of anaesthetized healthy C57BL6 wildtype mice. Afterwards we will prepare organotypic brain slices and examine, (1) whether platelets induce vessel damage, (2) activate matrix metalloproteinases (MMPs), (3) induce beta-amyloid-like immunoreactivity and (4) activate inflammatory processes including Iba1^+^ microglia.

## Results

### Flow Cytometry (FACS) analysis

**Figure 2:**
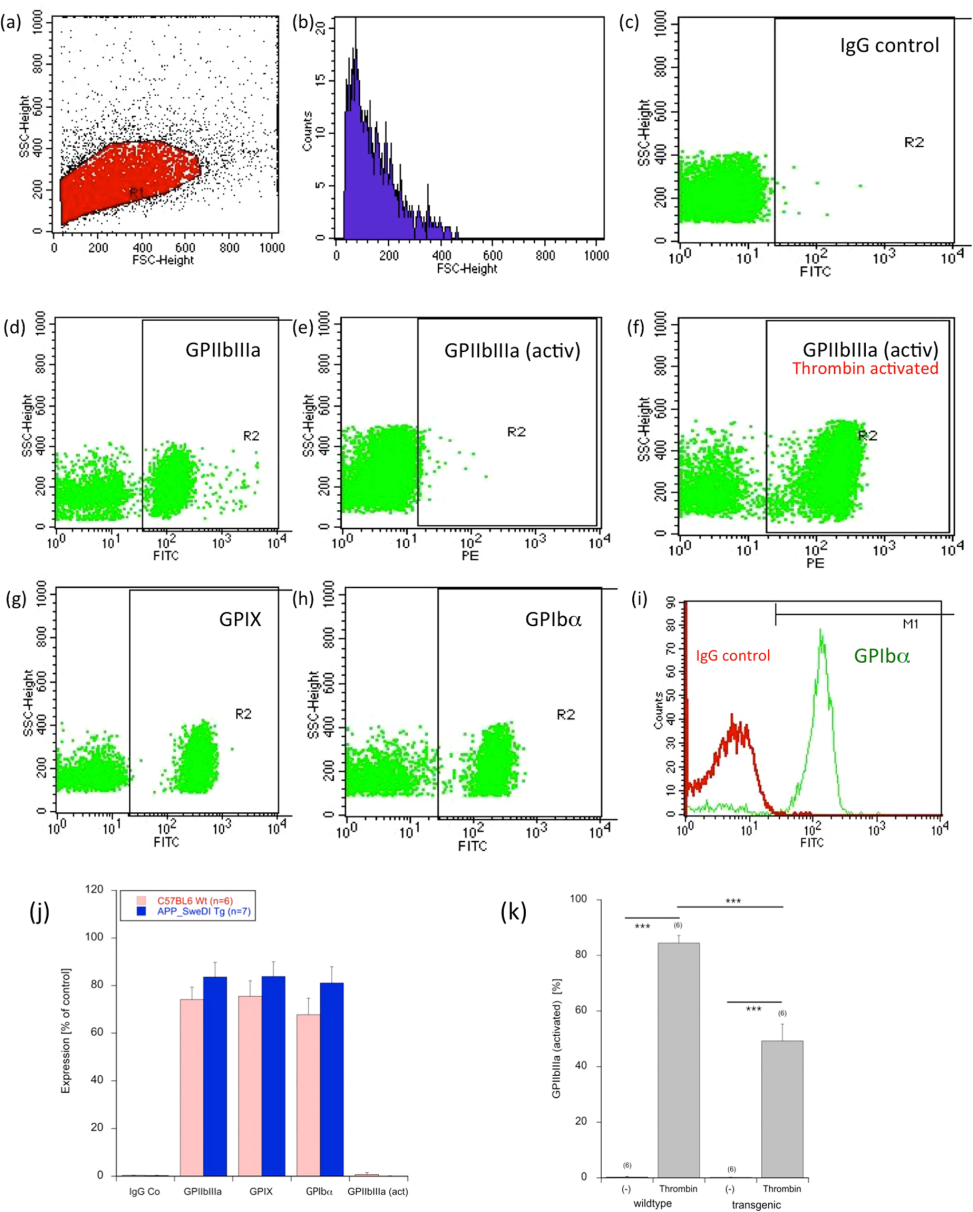
FACS analysis of isolated platelets. FACS analysis shows a single cell population in the forward versus sideward scatter plot (**a**) and in the counts vs. FSC blot (**b**). As a control always the respective IgG control was used, showing background staining (**c**). FACS analysis of isolated platelets shows a positive staining for GPIIbIIIa (CD41/CD61) (**d**), GPIX (CD42a) (**g**) and GPIbα (CD42b) (**h**). No staining was observed for activated GPIIbIIIa in untreated platelets (**e**), however, this was enhanced after thrombin activation (**f**). Figure (**i**) show again an IgG control versus the GPIbα. Quantitative analysis is given in Figure (**j**), showing no changes between wildtype or transgenic mice. However, the activation with thrombin induced GPIIbIIIa in wildtype as well as transgenic platelets. But platelets from transgenic mice were less sensitive for thrombin activation (**k**). Values are mean ± SEM (n = 6–7); statistical analysis was performed by students T-test (***p < 0.001).

In order to characterize activated platelets, FACS analysis shows a single cell population in the forward versus sideward scatter plot (Fig. 2a) and in the counts blot (Fig. 2b). As a control always the respective IgG control was used, showing background staining (Fig. 2c). FACS analysis of isolated platelets shows a positive staining for GPIIbIIIa (CD41/CD61) (Fig. 2d), GPIX (CD42a) (Fig. 2g) and GPIbα (CD42b) (Fig. 2h). No staining was observed for activated GPIIbIIIa in untreated platelets (Fig. 2e). However, this was enhanced after thrombin activation (Fig. 2f). Figure 2f shows an IgG control versus GPIbα. Quantitative analysis shows no changes between wildtype and transgenic mice (Fig. 2j). Interestingly, platelets from transgenic mice were less sensitive for thrombin activation (Fig. 2k).

### Infusion of PKH26^+^ platelets isolated from wildtype mice

**Figure 3:**
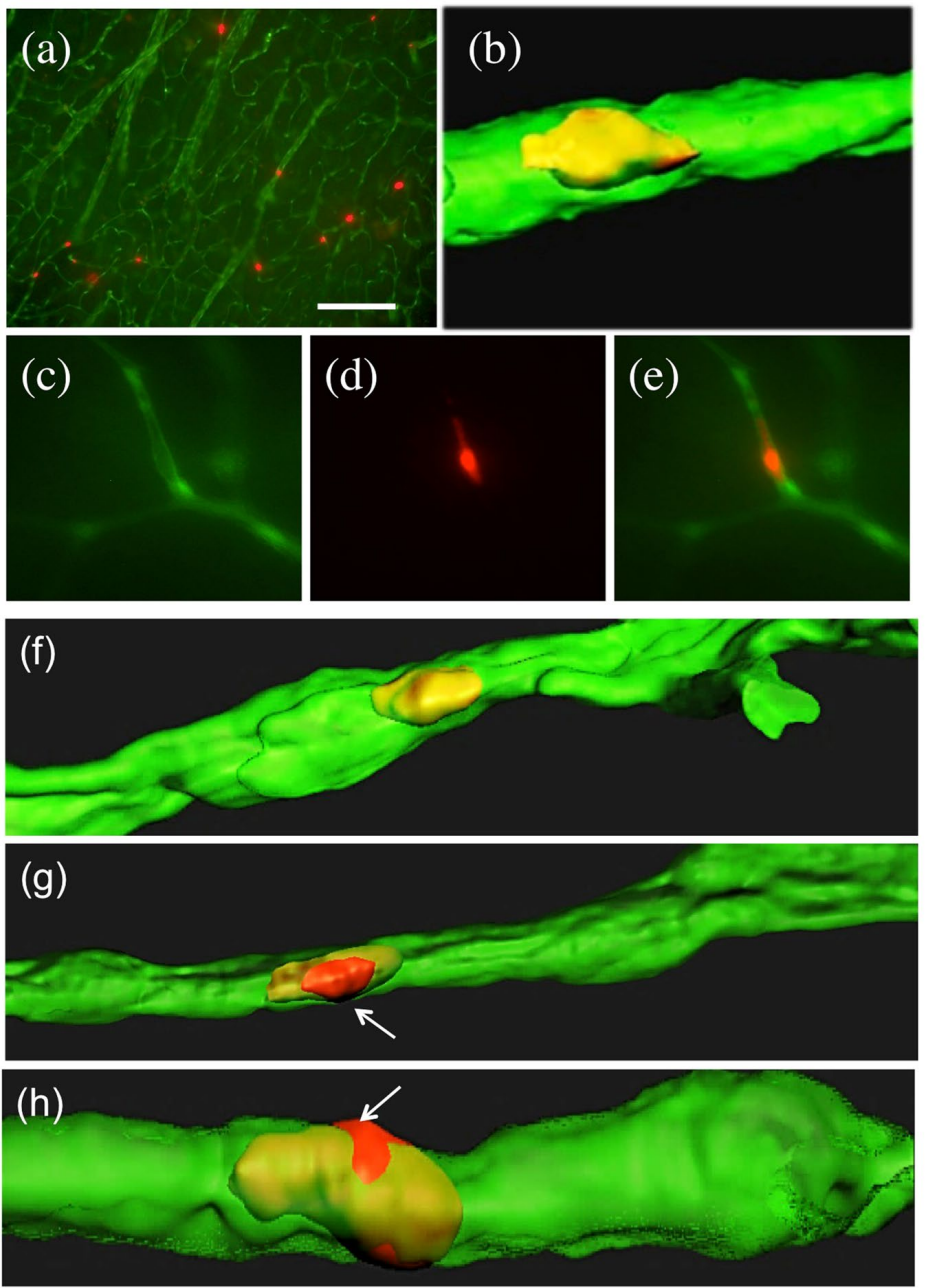
Infusion of PKH26 labeled platelets isolated from wildtype mice (**a**–**f**) or transgenic Alzheimer mice (**g**,**h**) into healthy C57BL6 wildtype mice. Freshly isolated PKH26 labeled platelets were slowly infused transcardially into wildtype mice, brains taken, sectioned with a vibratome into 110 µm thick organotypic brain slices, and cultured for 2 weeks, and counterstained with collagen IV. Collagen IV Alexa-488 was visualized in the green channel (EX 480/40 nm, EM 527/30 nm) and the red PKH26 platelets in the red channel (EX 535/50, EM 610/75) (**a**,**c**–**e**). Confocal images were performed with an argon laser line for Collagen IV Alexa-488^+^ vessels and a DPSS561 nm laser for PKH26^+^ platelets (**b**,**f**–**h**). PKH26 (red) labeled platelets isolated from wildtype mice are found in collagen IV (green) vessels (**a**,**c**–**e**), but they do not damage and penetrate the healthy vessel (**b**,**f**). However, platelets isolated and infused from transgenic Alzheimer mice into wildtype mice damage and penetrate the healthy vessel (**g**,**h**, arrows). Scale bar in **a** = 115 µm (**a**), 5 µm (**b**,**f**–**h**), 29 µm (**c**–**e**).

In the first set of experiments the effects of platelets isolated from wildtype mice were explored. When PKH26^+^ platelets isolated from wildtype mice were transcardially infused into adult C57BL6 wildtype mice several red platelets were seen in 110 µm thick organotypic brain slices after 2 weeks in culture (Fig. 3a). These platelets fully co-localized with green collagen IV Alexa-488 brain vessels (Fig. 3b,f). The microscopic evaluation was specific, because the green vessels were only visible in the green channel (EX 480/40 nm, EM 527/30 nm) (Fig. 3c), PKH26^+^ platelets only in the red channel (EX 535/50, EM 610/75) (Fig. 3d) and completely co-localized (Fig. 3e). Confocal microscopy clearly showed that platelets were found within the green vessels (Fig. 3b) and did not penetrate through the vessels in any case (Table 1). The number of PKH26^+^ platelets in a field of 10 vessels was approx. 7 (Table 1), the volume per platelet was approx. 50 µm^3^ (Table 1) and the volume per vessel was approx. 4000 µm^3^ (Table 1) and not different on day 1 or 14 of culture. No platelets penetrated the vessels at all (Table 1).

**Table 1:** Quantification of infused PKH26^+^ platelets in cortical brain vessels and effects of the MMP inhibitor.

		WT	TG	TG + MMP INH
Platelets/10 vessels	+1d	6.2 ± 0.8	7.6 ± 0.7 ns	7.3 ± 0.7 ns
	+7d	na	7.5 ± 1.2 (3)	na
	+14d	7.0 ± 0.7	7.7 ± 0.3 ns	7.5 ± 0.6 ns
Volume vessel [µm^3^]	+1d	2173 ± 423	3705 ± 489*	3354 ± 122 ns
	+7d	na	4001 ± 989 (3)	na
	+14d	4041 ± 419	4479 ± 814 ns	3381 ± 131 ns
Volume platelet [µm^3^]	+1d	43 ± 17	202 ± 37**	82 ± 8^#^
	+7d	na	112 ± 87 (3)	na
	+14d	50 ± 9	75 ± 17*	75 ± 3 ns
Platelet penetration per 10 vessels	+1d	0	0.5 ± 0.2	0.2 ± 0.1
	+7d	na	2.3 ± 1.9 (3)	na
	+14d	0	7.0 ± 3.1*	0^##^
Penetration from vessel [µm]	+1d	0	1.5 ± 0.6	0.1 ± 0
	+7d	na	1.8 ± 1.2 (3)	na
	+14d	0	2.1 ± 0.9**	0^##^

Platelets were isolated from 12-months old C57BL6 mice (WT, n = 6) or APP_SweDI (TG, n = 6) mice, labeled with the red fluorescent dye PKH26 and transcardially infused into anaesthetized 6-months old C57BL6 healthy wildtype mice. Brains were taken, 110 µm thick organotypic brain slices were made and cultured for 1 or 14 days. Brain sections were postfixed and counterstained for collagen IV (Alexa-488). In an additional experiment (n = 6) the matrix metalloproteinase inhibitor I (CAS 1177749-58-4; MMP INH) was added during isolation and infusion of platelets and incubation of brain slices. At a 10x magnification the number of cortical vessels and PKH26 labeled platelets was counted under the fluorescence microscope. Confocal microscopy was performed at a 63x magnification and the volumes of vessels and platelets were measured, as well as the penetration of platelets from the vessels. Three confocal pictures were analyzed per brain. Values are given as mean ± SEM. Statistical analysis was performed by One Way ANOVA with a Fisher LSD posthoc test where TG were compared against WT (*p < 0.05; **p < 0.01; ns not significant) or against TG + MMP INH (^##^p < 0.01). For the 7 day cultures only the Tg were evaluated with n = 3 (na, not analyzed).

### Infusion of PKH26^+^ platelets isolated from APP_SweDI mice

In the second set of experiments the effects of platelets isolated from transgenic AD mice were tested and compared to the platelets isolated from wildtype mice. When freshly isolated platelets from transgenic APP_SweDI Alzheimer mice were infused into adult C57BL6 wildtype mice and cultured for 2 weeks, the most pronounced and visible effect was that platelets damaged the vessel wall and penetrated through the wall extending into the extracellular space (Fig. 3g,h). In no case this damage was seen when platelets from wildtype mice were infused (Fig. 3b). In average 7 platelets penetrated the vessel wall out of 10 counted vessels (Table 1). The average penetration into the brain parenchyma was approx. 2.1 µm (Table 1). The volume of platelets was significantly increased from AD mice compared to wildtype mice (Table 1). Neither the number of platelets/10 vessels, nor the volume of the vessel was different to wildtype infused platelets (Table 1). The number of penetrations significantly increased from 1 to 14 days (Table 1).

### Effects of the MMP inhibitor I

In order to block any MMP-mediated effects on platelet penetration a MMP-inhibitor was tested. When the MMP inhibitor was added to platelets derived from transgenic mice during isolation, transcardial infusion and incubation of the organotypic brain slices, the disruption of cortical vessels caused by transgenic platelets was completely abolished (Table 1). Neither the number of platelets/10 vessels, nor the volume of platelets or vessels was different (Table 1).

### Co-staining of platelet markers CD41 and CD61 with PKH26 platelets

**Figure 4:**
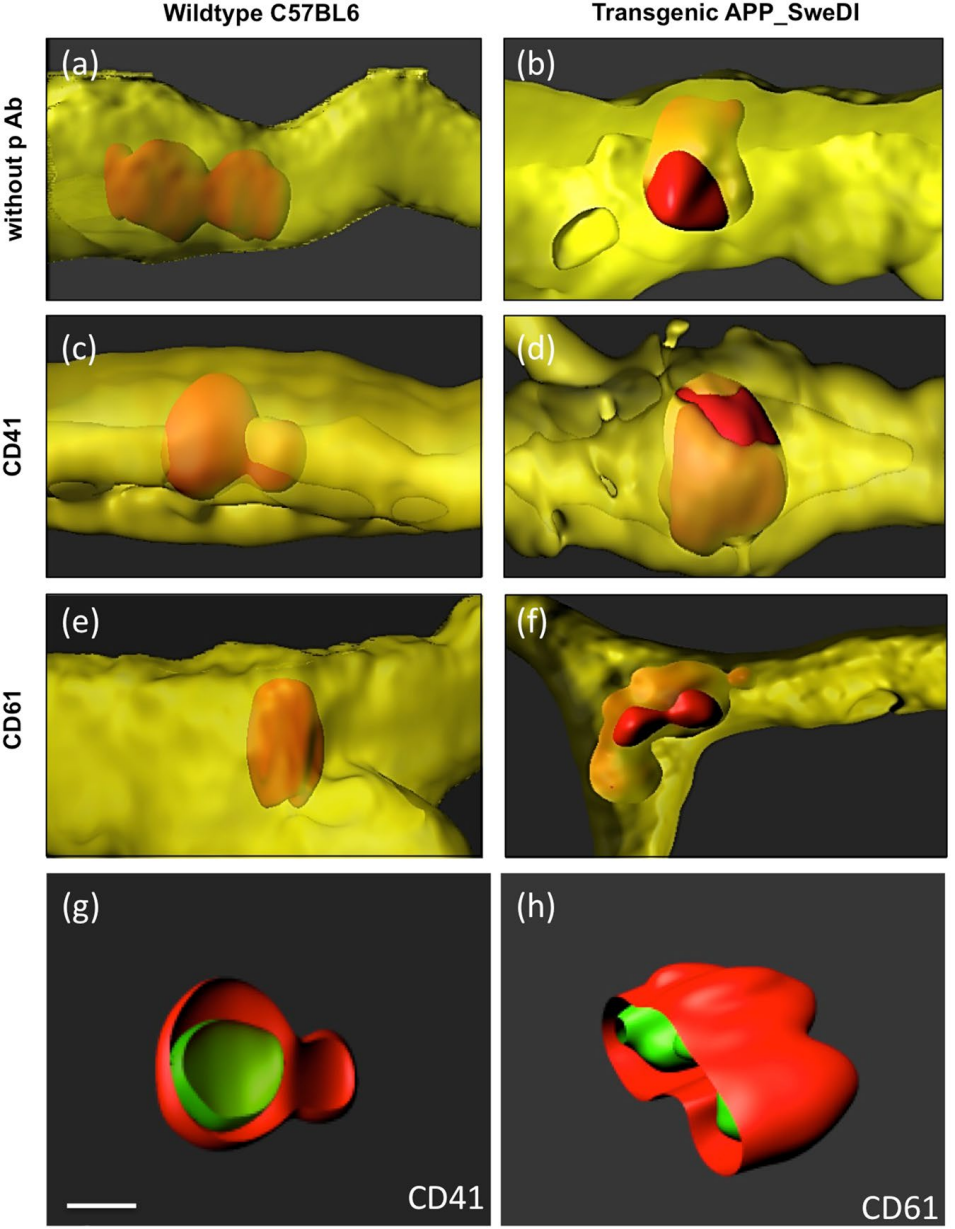
Co-localization of infused (red fluorescent) PKH26^+^ platelets with selective (green fluorescent) platelet markers CD41 (**c**,**d**,**g**) and CD61 (**e**,**f**,**h**). Platelets were isolated from wildtype mice (**a,c,e**) or transgenic Alzheimer mice (**b**,**d**,**f**–**h**), labeled with the red fluorescent dye PKH26, transcardially infused, organotypic brain slices prepared, incubated for 2 weeks and then counterstained with the vessel marker collagen IV (Alexa-647, shown in yellow) or with the CD41/CD61 (Alexa-488, shown in green). As a negative control the primary antibody was omitted (**a**,**b**) showing no positive green CD41/CD61 staining but the red fluorescent PKH26^+^ platelets. Note that red platelets only penetrated vessels when isolated from transgenic Alzheimer mice (which is clearly seen in Figures (a) versus (b)). The platelet markers CD41 and CD61 fully co-localized with the red PKH26^+^ platelets, showing that the PKH26^+^ platelets display intracellular CD41 as well as CD61 immunoreactivity (**g**,**h**). Scale bar in **g** = 5 µm (**a**–**h**).

In order to test that the PKH26^+^ platelets are not affected by the membrane labelling, a co-staining with the platelet specific markers CD41/CD61 was performed. Co-stainings were performed using collagen IV (Alexa-647), red PKH26^+^ platelets and the respective markers (Alexa-488). Immunostainings revealed that both platelet markers CD41 and CD61 fully co-localized with the PKH26^+^ red labeled infused platelets, in wildtype as well as transgenic mice (Fig. 4c–f ). As a negative control the primary antibody was omitted (Fig. 4a,b) showing no positive green stainings. Figure 4g,h show that CD41 and CD61 fully co-localized intracellular with the platelets.

### Vessel damage caused by platelets isolated from AD mice

**Figure 5:**
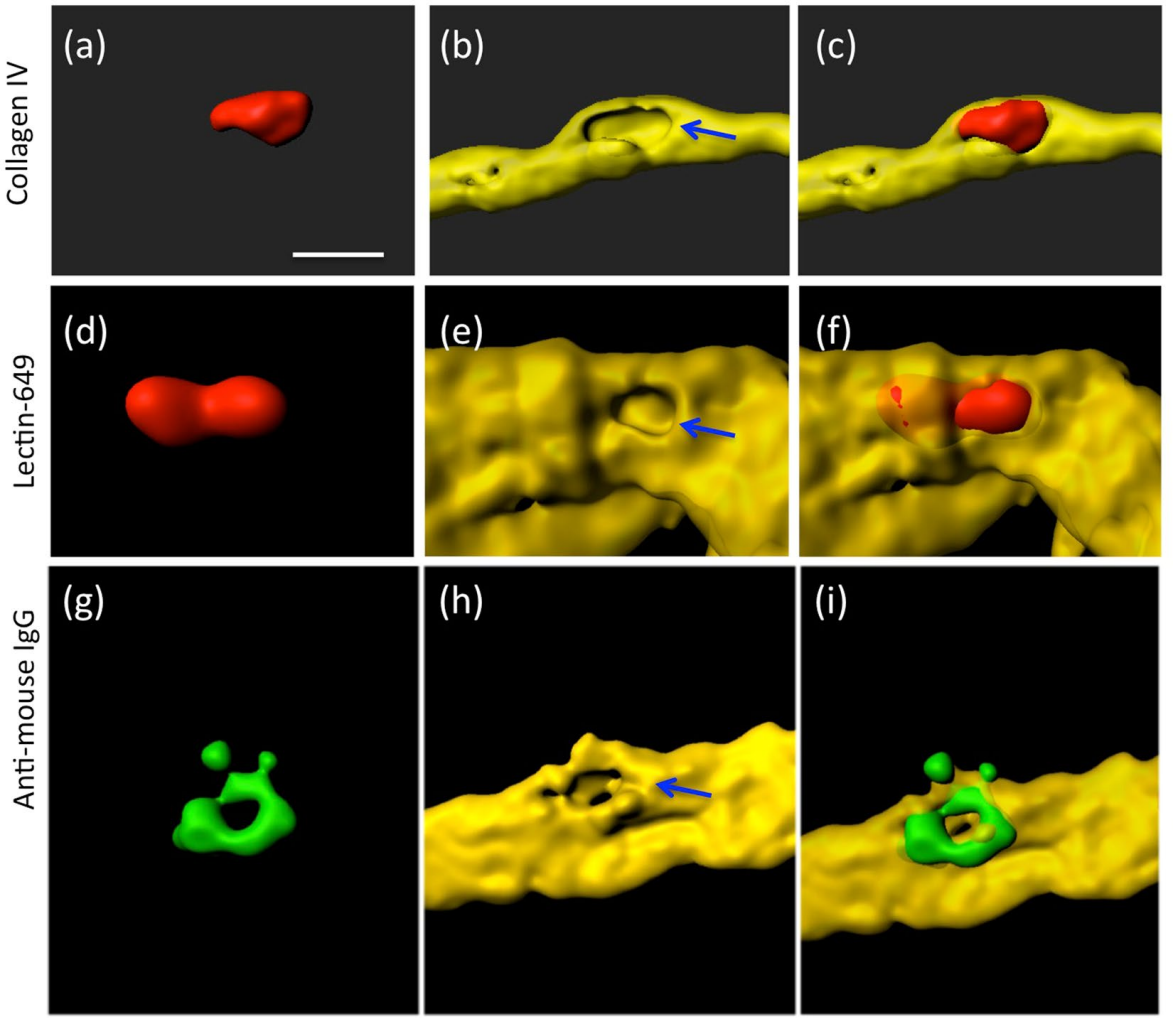
Vessel damage caused by transcardial infusion of red PKH26 platelets (**a**,**d)** isolated from transgenic Alzheimer mice. Vessels were stained with Collagen IV (Alexa 647, (**b**) or Lectin-649 (**e**,**h**). Merged pictures showing red penetrating platelets in vessels are given in Figure (**c**,**f**). Staining for anti-mouse IgG (Alexa-488; **g**) shows vessel damage. Note several holes (arrows in **b**,**e**,**h**) in the vessel, where the platelet penetrates the vessel, indicating vessel damage. Scale bar in **a** = 7 µm (**a**–**i**).

Lectin is a very simple to use dye (only 1 hour incubation) and superior to the collagen IV immunostainings (over 2 days). Thus a comparison was made between the 2 vessel markers Lectin and collagen IV. The Supplementary Fig. 1 shows almost entire co-localization of Collagen IV and Lectin-649, whereupon vessel staining was performed with Lectin-649 as it provides a much faster and easier method of vessel staining. These stainings were combined with anti-mouse IgG in order to show if the vessels are damaged and if blood-derived IgG is found. In both stainings immunoreactive negative holes were visible at the site where platelets penetrated the vessel (Fig. 5a–f). Similarly anti-mouse IgG-Alexa-488 pointed to blood-brain barrier damage in vessels (Fig. 5g–i).

### MMP immunostainings and release

**Figure 6:**
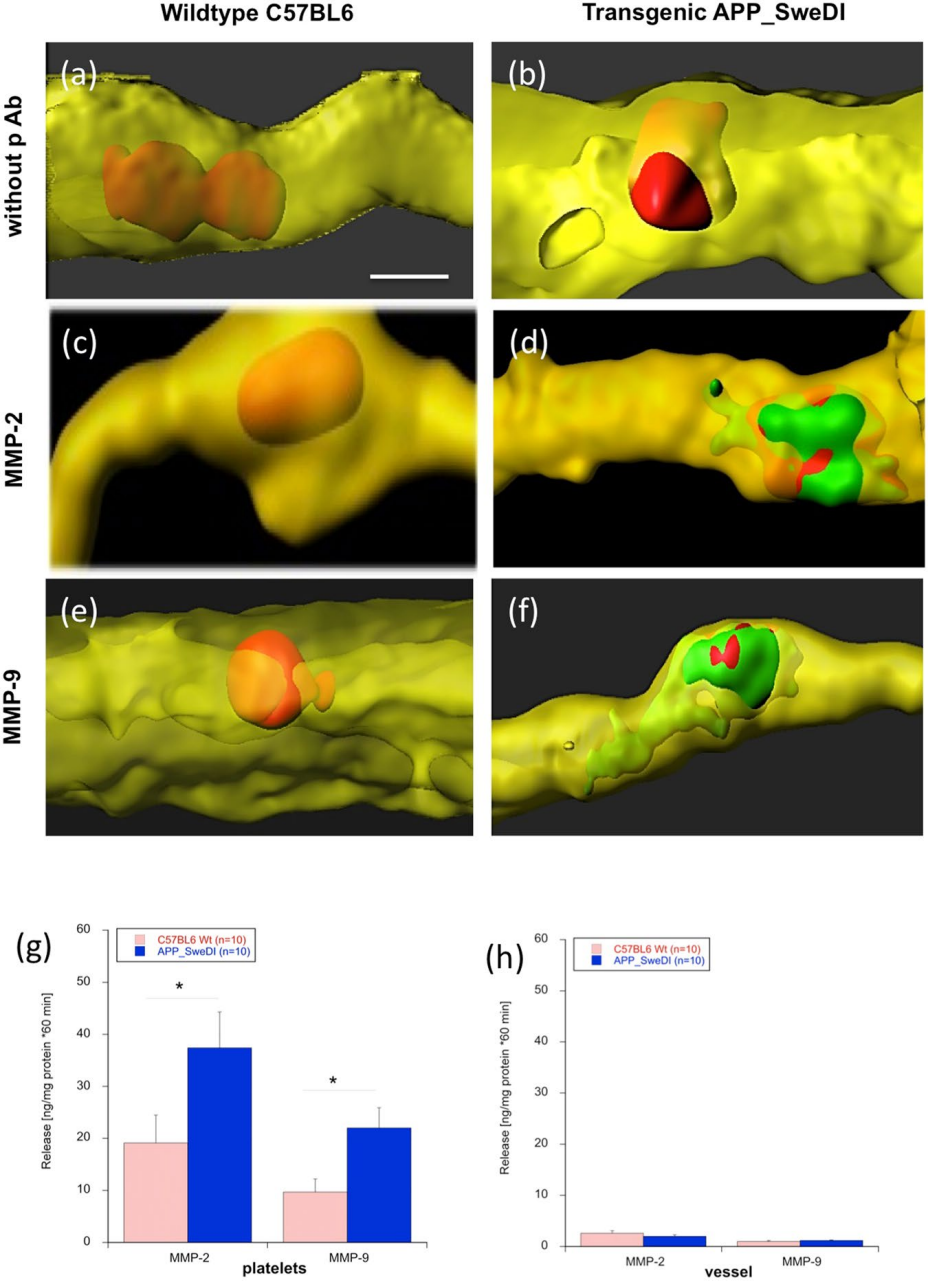
Matrix metalloproteinases (MMPs) in vessels. Platelets were isolated from wildtype mice (**a**,**c**,**e**) or transgenic Alzheimer mice (**b**,**d**,**f**), labeled with the red fluorescent dye PKH26, transcardially infused, organotypic brain slices prepared, incubated for 2 weeks and then counterstained with Lectin-649 (shown in yellow) or with the MMP-2 or MMP-9 antibodies (Alexa-488, shown in green). As a negative control the primary antibody was omitted (**a**,**b**) showing no positive green staining. Note that red platelets only penetrated vessels when isolated from transgenic Alzheimer mice **(b**,**d**,**f**). Note that MMP-2 and MMP-9 were expressed only when platelets penetrated the vessel. Scale bar in (**a**) = 5 µm (**a**–**f**). In order to show release of MMP-2 and MMP-9, platelets (**f**) or cortical vessels (**h**) were isolated from wildtype or transgenic mice, incubated for 60 min and the levels of MMP-2 and MMP-9 measured by ELISA. Values are given as mean ± SEM (n = 10). Statistical analysis was performed by students T-Test (*p < 0.05). Note significant more release of MMP-2 and MMP-9 from platelets (**g**) isolated from transgenic mice but not from vessels (**h**).

In order to show that the platelet penetration and vessel damage involves matrix-metalloproteinases an immunostaining was performed. Immunostainings clearly showed increased MMP-2 and MMP-9 around platelets, which penetrated through the vessels (Fig. 6d,f), but not in the non-penetrating vessels (Fig. 6c,d). Again omission of the primary antibody showed only background (Fig. 6a,b). In order to demonstrate release of MMPs from platelets (Fig. 6g) or cortical vessels (Fig. 6h), ELISA assays were performed. Platelets isolated from AD mice significantly release more MMP-2 and MMP-9 compared to platelets isolated from WT mice (Fig. 6g). No release of MMP-2 and MMP-9 was seen from cortical vessels (Fig. 6h).

### Beta-amyloid, Thiazine Red and Resorufin

**Figure 7:**
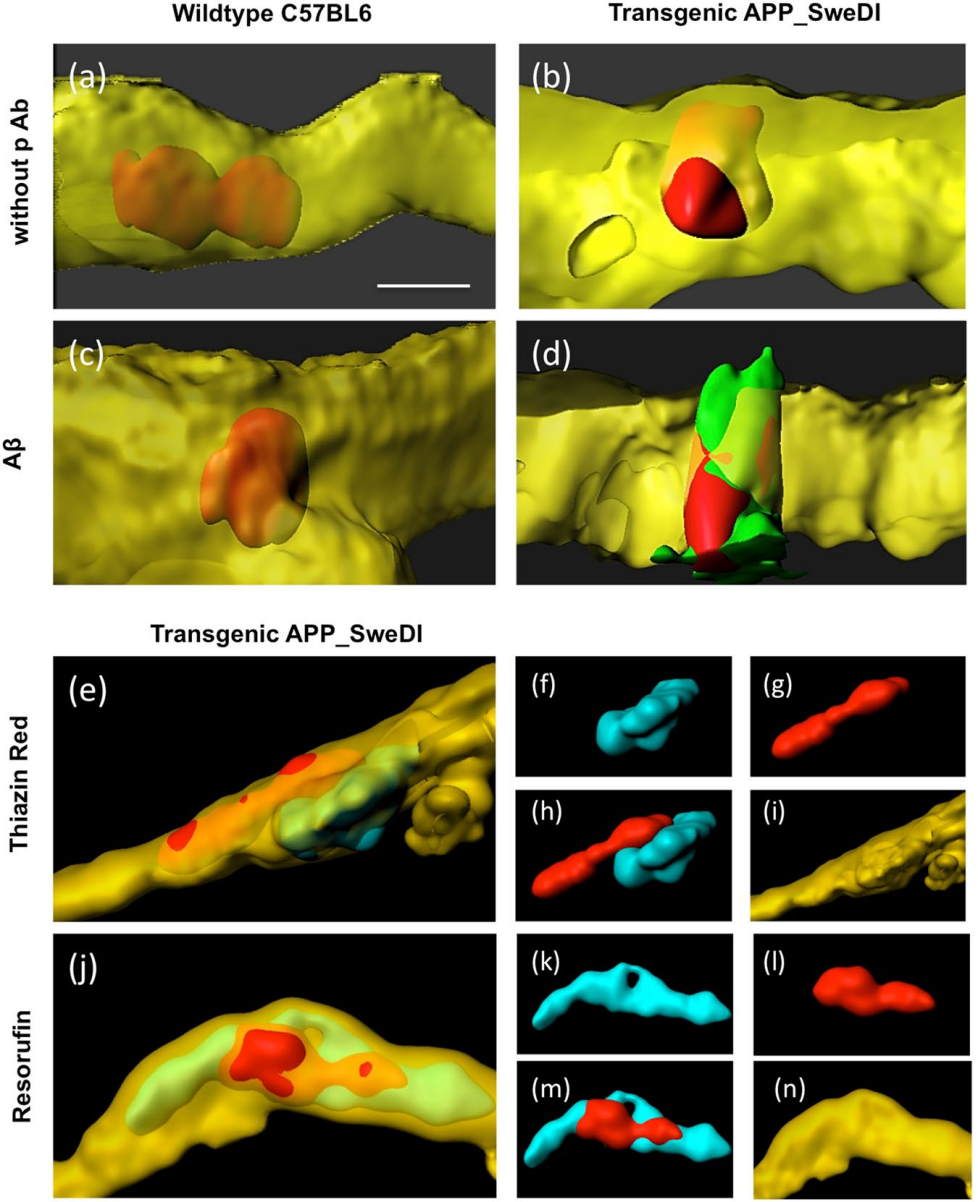
Beta-amyloid, Thiazine Red and Resorufin stainings in vessels. Platelets were isolated from wildtype mice (**a**,**c**) or transgenic Alzheimer mice (**b**,**d**,**e**–**n**), labeled with the red fluorescent dye PKH26, transcardially infused, organotypic brain slices prepared, incubated for 2 weeks and then counterstained with the vessel marker Lectin-649 (shown in yellow). Immunostainings show beta-amyloid-like immunoreactivity (**c**,**d**), or Thiazine Red (**e**–**i**) or Resorufin (**j**–**n**). As a negative control the primary antibody was omitted (**a**,**b**) showing no positive green staining but still the red fluorescence PKH26 platelets. Note that red platelets only penetrated vessels when isolated from transgenic Alzheimer mice (**b**,**d**,**e**,**j**). Note beta-amyloid immunoreactivity, Thiazine Red and Resorufin around vessels with penetrations from platelets isolated from transgenic mice (**d**,**e**,**j**). Figure **(f**–**g** and **k**–**n)** show the single channels of the respectives Figures (e) and (j). Scale bar in **a** = 5 µm (**a**–**d**), 10 µm (**e**,**j**), 25 µm (**f**–**i**, **k**–**n**).

In order to show if the platelet penetration is accompanied by enhanced beta-amyloid expression, immunostainings were performed and compared to the cytoplasmic Thiazine Red and vascular Resorufin stainings. Co-stainings revealed pronounced immunostaining for Aβ when platelets were infused from AD mice (Fig. 7d) but not from WT mice (Fig. 7c). Omission of the primary antibody again showed a negative staining (Fig. 7a,b). Thiazine Red inclusions were clearly visible in vessels isolated from AD mice (Fig. 7e–i). Similarly, Resorufin co-staining showed a clear staining located next to the platelets around the vessel walls (Fig. 7j–n).

### Inflammatory processes

**Figure 8:**
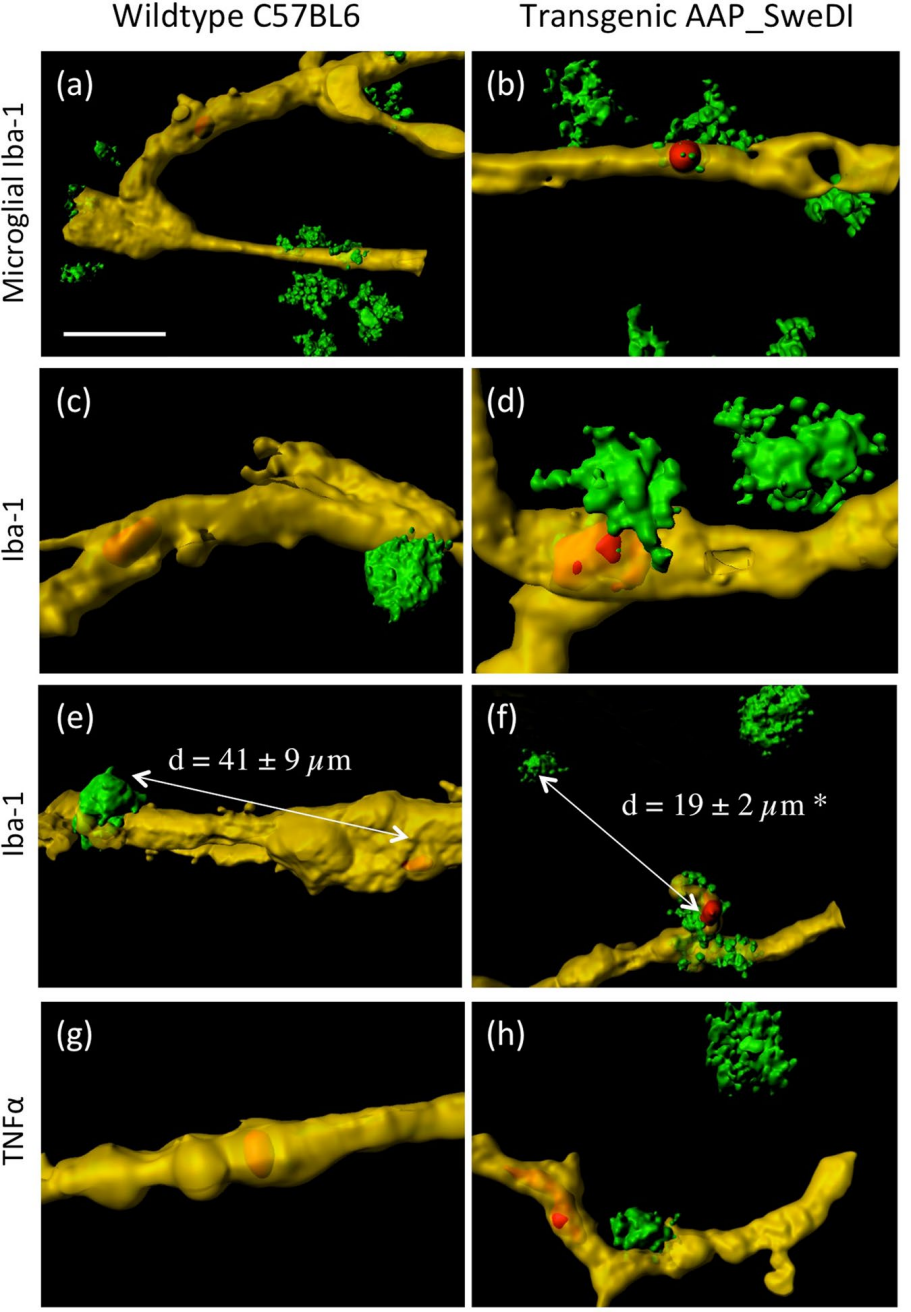
Inflammatory responses around vessel penetrating platelets. Platelets were isolated from wildtype mice (**a**,**c**,**e**,**g**) or transgenic Alzheimer mice (**b**,**d**,**f**,**h**), labeled with the red fluorescent dye PKH26, transcardially infused, organotypic brain slices prepared, incubated for 2 weeks and then counterstained with Lectin-649 (shown in yellow). Microglial cells were stained with Iba-1 (**a**–**f**) or tumor-necrosis-factor-alpha (**g**,**h**), both with Alexa-488 (green). Note that red platelets only penetrated vessels when isolated from transgenic Alzheimer mice. Note that the distance between platelets and microglial cell was significantly (p < 0.05) reduced when platelets penetrated the vessel (41 ± 9 µm in WT versus 19 ± 2 µm in TG mice, n = 10, (**e**,**f**). Scale bar in **a** = 15 µm (**a**,**b**,**e**,**f**) and 5 µm (**c**,**d**), and 10 µm (**g**,**h**).

In order to visualize inflammatory processes due to the vessel damage and platelet penetrations, microglial Iba1^+^ co-stainings were performed and the cytokine TNF-α was analyzed. Iba1^+^ microglia were clearly seen around the Lectin-649^+^ vessels after transcardial infusion of platelets isolated from WT (Fig. 8a,c,e) and TG (Fig. 8b,d,f) mice. The distance between platelets and Iba-1^+^ microglial cells was significantly reduced when platelets penetrated the vessel (19 ± 2 µm, n = 10) compared to the wildtype platelets (41 ± 9 µm, n = 10) (Fig. 8e,f). The proinflammatory cytokine TNFα, was only apparent near penetrated vessels while in wildtype platelets TNFα was absent (Fig. 8g–h).

## Discussion

**Figure 9:**
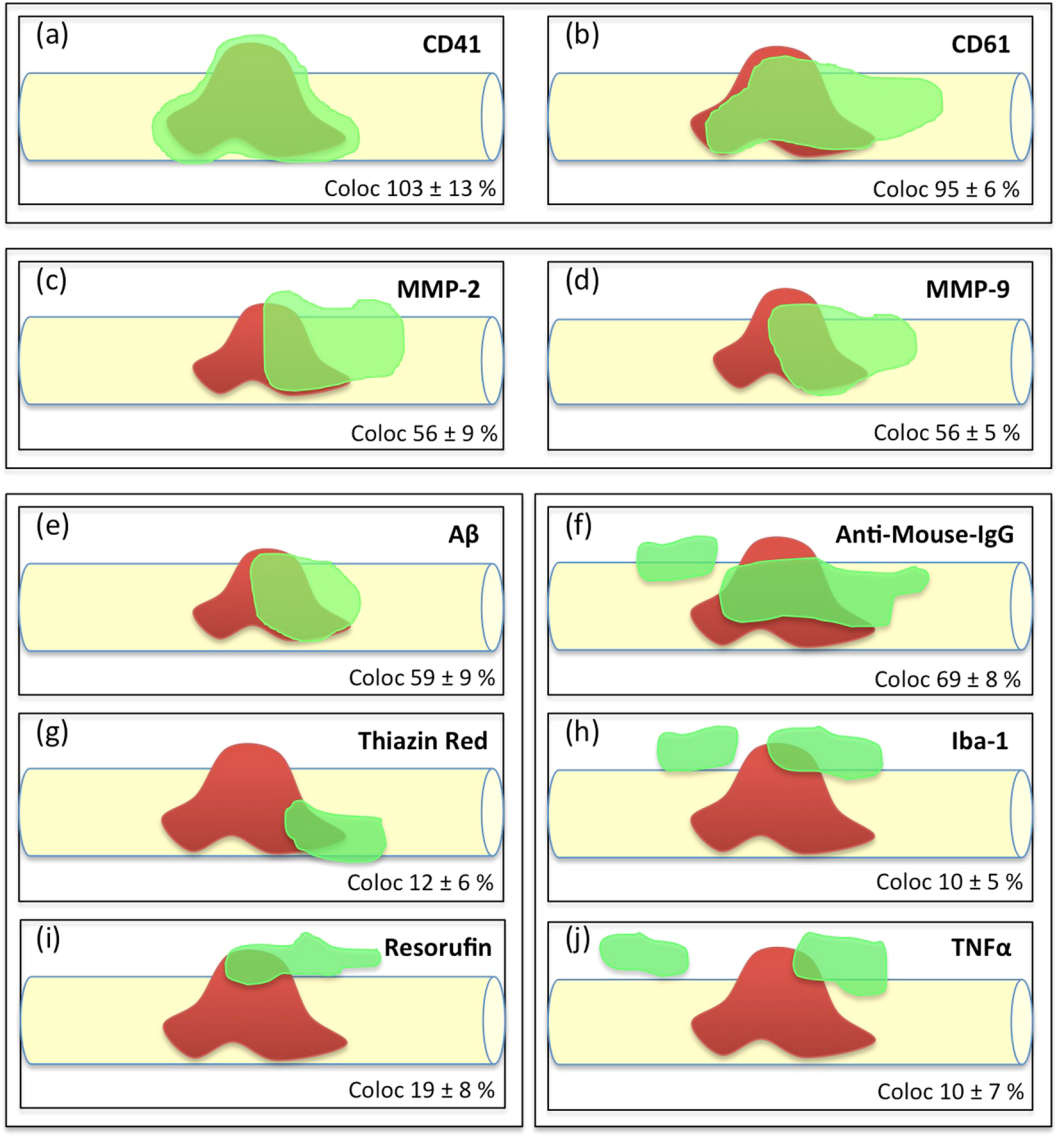
Cellular processes around platelet penetrations in a cortical vessel (schematic drawings). This scheme shows the putative processes around a platelet which penetrates through a vessel (taken from 10 representative confocal images). A penetrating platelet is shown in red in a yellow vessel and the co-markers are given in green. Our data reveal (1) that the platelet markers CD41 and CD61 fully co-localize with PKH26 red platelets (**a**,**b**), (2) that matrix metalloproteinases −2 and −9 are activated and released possibly playing a role in vessel damage (**c**,**d**), (3) that platelets produce and release beta-amyloid, which is also stained by Thiazine Red and Resorufin (**e**–**i**) and (4) that the platelet penetration causes inflammatory processes, as seen by influx of mouse IgG (**f**), microglial activation and migration (**h**) and enhanced expression of tumor-necrosis factor-alpha (**j**).

In the present study we aim to explore if platelets isolated from AD mice (APP_SweDI) damage healthy cortical brain vessels. In order to study this issue we isolated platelets from AD (APP_SweDI) and wildtype (C57BL6) mice, labeled them with the red fluorescent dye PKH26 and transcardially infused the freshly isolated and labeled platelets into healthy C57BL6 wildtype mice. In cultured organotypic brain slices we show that platelets isolated from AD mice damage healthy cortical vessels, activate MMPs, induce beta-amyloid like immunoreactivity and neuroinflammation. All results are summarized in a scheme (Fig. 9) showing the cellular processes around a vessel penetrating platelet.

### Stimulated PKH26^+^ platelets in AD

Platelets play an important role during the progression of AD as discussed previously^14,24^. We have extensive experience in isolation of mouse and human platelets and fully characterized the cells by FACS^23^. We also have well established the fluorescence labelling of platelets using the dye PKH26^25^. The fluorescent dye PKH26 binds mainly to the cell membrane, has a strong membrane retention and gives strong fluorescent emission as compared with other fluorescent dyes^26^. Indeed we show that nearly 100% of the PKH26 fluorescence co-stains with the specific platelet markers CD41 or CD61. Previously we also studied the migration of platelets into the brain after *in vivo* tail vein infusion and observed that the red dye PKH26 is very useful for *in vivo* cell tracking^25^. Recently, we reported that Thiazine Red^+^ platelets are present in the brain of young AD mice, representing a first sign of AD progression before Aβ plaques become evident^23^. We hypothesize^24^ that early stimulation of platelets may play a role in the development of AD, especially in the progression of CAA. Thus, in the present project we isolated platelets from AD and wildtype mice, labeled these platelets with the dye PKH26 and observed the effects on healthy cortical vessels in a novel organotypic infusion brain slice model. We found that the volume of AD-derived platelets significantly increased (approx. 75 µm^3^) compared to wildtype-derived (approx. 50 µm^3^) platelets. The platelet volume is indeed a marker of platelet activation and an indication for vascular events linked to AD. However, the volume of platelets in AD is very divergent, where some reported increased and others decreased platelet volumes, which may point to heterogenous apoptotic processes^18^. We further show using phenotypic FACS analysis that the platelets isolated from AD mice are indeed different to WT mouse platelets, as seen by a reduced sensitivity to thrombin. Finally, AD-derived platelets release an enhanced amount of MMPs. This all strengthens the fact, that platelets from AD mice are in a stimulated form.

### The novel infusion organotypic brain slice model

To test the hypothesis that AD-derived platelets damage healthy cortical vessels, we developed a novel *in vitro* model. As discussed, we already tested *in vivo* infusion of platelets via the tail vein into mice, however, most of the infused platelets were captured in the periphery (spleen, liver, lung) and only a few platelets entered the brain^25^ and thus such an *in vivo* model is not useful. As we have long lasting experience with organotypic brain slices^26^, we developed this novel *in vitro* infusion slice model. We first isolated platelets from mice, labeled them with the red fluorescent dye PKH26, and afterwards we transcardially infused these platelets immediately into anesthetized C57BL6 healthy control wildtype mice. This method was based on our long lasting experience of transcardial perfusions for fixation of brains using paraformaldehyde. Immediately after infusion the brains were taken, sectioned into 110 µm thick brain slices using a vibratome and checked under the fluorescence microscope (see also the Workflow Fig. 1). The method was optimized and a transcardial infusion of 3 ml platelet suspension resulted in approx. >10 fluorescent platelets per brain section. Sections with fewer platelets per section were not used, which can be caused by incomplete infusions. The selected slices were then transferred to 0.4 µm semipermeable membrane inserts and cultured for 1 and 14 days as organotypic brain slices as reported by us^26^. Although our model has been extensively evaluated and characterized, this brain slice model is still a stationary model lacking any blood flow, and thus the effects of platelet adhesion to vessels could be different in an *in vivo* situation.

### Platelets derived from AD mice damage healthy vessels

There is clear evidence that vessels are stimulated in the AD brain and these cerebrovascular changes are often accompanied by perivascular denervation^27^. Our data show for the first time that platelets isolated from AD mice but not from WT mice damage healthy cortical brain vessels, which is a first sign for the AD progression. This damage was visualized in a way that the platelets penetrated the vessel wall and entered the brain parenchyma, as this was shown in 3D confocal microscopy. The platelets entered the brain side within a distance of approx. 2 µm, which is very low and in no cases we observed that a whole platelet entered the brain side. However, as platelets have a half life time of 5–7 days it is possible that they lose function in the brain after 7–14 days cultured in brain slices.

Our data provide clear evidence that the platelet penetration caused vessel damage. This can be seen as clear immunoreactive-negative spots in the vessel using the vessel markers collagen IV and Lectin. Further, we show increased anti-mouse IgG directly around the vessel damage, pointing to efflux of blood-derived IgGs. Such IgG^+^ spots very likely correlate to the well known MRI^+^ microbleeds frequently seen in AD and in vascular dementia^28,29^.

### Platelets and MMP

Matrix Metalloproteinases (MMPs) are a group of approximately 40 homologous proteases and it is well known, that MMPs and their inhibitors contribute to a variety of pathophysiologies, including cell migration, tissue degradation or inflammation. MMPs can degrade the extracellular matrix and have been implicated in the blood-brain barrier breakdown in neurodegenerative diseases^30^. Interestingly, platelets synthesize and secrete a variety of MMPs^31–33^. Thus, platelet-associated MMP activity appears to play a major role in these processes. In human AD patients, we have already published that levels of MMP-2 are decreased in platelets possibly pointing to enhanced release during the AD progression^34^. Thus there are clear indications, that MMPs caused by vessel disruption may play a role in the vessel pathology during AD progression. In our present study we indeed show that MMP-9 and MMP-2 like immunreactivity is increased around the platelets in the lesioned vessel. In order to test if platelets or vessels release MMPs, we performed *in vitro* release experiments and show that exclusively platelets release MMPs and not vessels. Thus we favour the idea that the released MMPs come directly from platelets during vessel penentration. In order to confirm our results on MMPs, we added an MMP inhibitor during the whole experiment that means during isolation of platelets, during infusion into C57BL6 mice and also during culturing of brain slices. Our data clearly show that the MMP inhibitor completely counteracted the damage of the vessels and the penetration of AD-derived platelets. In our experiments we used the cell-permeable MMP inhibitor I (CAS 1177749-58-4, Calbochem) at a concentration where it blocks MMP-9, MMP-2 and MMP-13.

### Platelets penetrating vessels induce beta-amyloid stainings

As discussed previously, vascular alterations in AD are very frequent, including e.g. increased number of fragmented vessels, changed vessel diameters, altered capillary membranes or collagen accumulations in the basement membrane^35^. Thus this damage of brain vessels very likely activates platelets, which play a role in thrombosis and hemostasis at sites of vascular damage. In this respect it is noteworthy, that stimulated platelets aggregate at sites of vascular lesion, release Aβ and induce thrombus formation, which leads to vessel occlusion^1,36^. The role of platelet-derived Aβ is not fully clear, but there are indications that it may play a role as a clotting substance^16,37–39^. Our present data show, that AD-derived platelets not only damage healthy vessels, but also that beta-amyloid-like immunoreactivity is found around the damaged vessel walls. This is in full agreement with Donner *et al*.^40^, who showed that platelets contribute to Aβ aggregation in cerebral vessels involving clusterin. Our immunostainings clearly show Aβ-like immunoreactivity around penentrating vessels. In the present study we used an antibody which is reactive to amino acids 1–16 and thus recognizes all isoforms and precursor forms. It is very likely that Aβ is released from platelets, as this immunoreactivity is closely associated with the platelets as seen in confocal microscopy. These data are also strenghtened because we also show staining for intracellular Thiazine Red, which also stains plaques. In order to characterize CAA the phenoxazine derivative Resorufin has been tested which preferentially binds cerebrovascular amyloid^23^. Indeed, we verify vascular staining of Resorufin around platelets penetrating the damaged vessel. Thus, our data show for the first time that AD-derived platelets are involved in the enhanced expression of beta-amyloid in the damaged vessel wall. It is thus very likely that this process may play an initial role in the progression of CAA.

### Platelets penetrate vessels and cause inflammation

It is well known that the brain employs various immune control mechanisms to regulate inflammatory processes as well as brain disruption pathways. Increased vascular permeability along with blood flow at the site of injury or inflammation are typical features during an immune response in AD accompanied by the entry of immune cells from the periphery^41–43^. Indeed, our data provide evidence that microglia became activated due to vessel damage and platelet penetration. More importantly, the activated Iba-1^+^ microglia migrated to the penetrated vessels in the damaged vessels and were located significantly closer to the damaged vessel sites compared to the wildtypes. This was verified by measuring the mean distance between the Iba1^+^ microglia and the next platelet in the vessels, where we show that the activated microglia migrated to the vessel penetrating platelets. This clearly points to an immune reaction caused by platelet-derived vessel damage.

Furthermore, platelets adhere to the vascular lesion site, accumulate and secrete various cytokines, contributing to tissue repair^44^. The rationale to measure tumor-necrosis-factor-alpha (TNF-α) was a report linking TNF-α to MMP9 in disruption of the blood-brain barrier^45^. Indeed, our data clearly show enhanced TNF-α immunoreactivity around the damaged vessel close to platelet penetrations. TNF-α could be either detrimental in a way to induce apoptosis of damaged microvascular cells or there is also evidence that TNF-α mediates microvascular repair processes^45^.

#### Suggested mechanistic processs

Our present data strengthen our hypothesis^24^ that stimulated platelets may play a role in progression of CAA and possibly AD. We suggest that vascular risk factors cause vessel damage with subsequent influx of toxic blood-derived IgGs or immune cells^46^ over decades in humans. This vessel damage continously activates platelets, which undergo an altered status and produce and generate MMPs. In order to repair vessel damage, platelets produce excess of Aβ to clot the leakage, which comes out of control over time causing uncontrolled Aβ release and function. Subsequently microglia become activated, migrate to the lesion sites and release pro-inflammatory TNFα in order to phagocyte and eliminate plaques. In summary, our data reveal (1) that the platelet markers CD41 and CD61 fully co-localize with PKH26 red platelets (Fig. 9a,b), (2) that matrix metalloproteinases −2 and −9 are activated and released possibly playing a role in vessel damage (Fig. 9c,d), (3) that platelets produce and release beta-amyloid, which is also stained by Thiazine Red and Resorufin (Fig. e–i) and (4) that the platelet penetration causes inflammatory processes, as seen by influx of mouse IgG (Fig. 9f), microglial activation and migration (Fig. 9h) and enhanced expression of tumor-necrosis factor-alpha (Fig. 9j).

In conclusion, we show that platelets isolated from AD mice damage healthy cortical brain vessels, which involve MMPs and lead to inflammatory processes. This vessel disruption is accompanied by increased beta-amyloid production around damaged vessels. Our data may suggest that anti-platelet drugs or MMP inhibitors could become useful therapeutic targets in counteracting the vessel damage and thus CAA during the progression of the AD pathology.

## Methods

### Mouse models

The Alzheimer mouse model (C57B1/6-[Thy1-APPSweDuIowa] Bwevn/Mmjax, APP_SweDI) was obtained from MMRRC (USA). These transgenic mice (TG) express neuronally derived human amyloid beta-precursor protein (APP 770 isoform) with the Swedish K670N/M671L, Dutch E693Q and Iowa D694N mutations (APP_SweDI). This model has been fully characterized and exhibits marked Aβ plaques in brain and vessels after 6 months of age^47^. As a control and for the infusion experiment adult C57BL/6 N wildtype (WT) mice were used. All animal experiments were approved by the Austrian Ministry of Science and Research (BMWF-66.011/0044-II/3b/2011 and BMWF-66.011/0059-II/3b/2011) and conformed to the Austrian guidelines on animal welfare and experimentation. All possible steps were taken to reduce suffering and the number of animals used during the experiments.

### Isolation and PKH26 labeling of platelets

The complete workflow of the experiment including the time flow is given in Fig. 1. Platelets were isolated as reported previously and labeled with the red dye PKH26 Red Fluorescent Cell Linker Kit (Sigma) as described by us^25^. Platelets were isolated by cardiac puncture, as this method does not cause activation of platelets^48^. Briefly, blood was taken from adult anesthetized (Ketamine 100 mg/kg and Xylazine 10 mg/kg (AniMedica) 12-months old C57BL/6 N mice or APP_SweDI mice. The blood was directly drawn from the heart and collected in EDTA tubes. Subsequently, the blood was centrifuged at 100 × g for 10 min at room temperature (RT) to obtain the platelet rich plasma (PRP). PGI_2_ (Prostaglandin, 500 nM, Sigma) was added and platelets were isolated from PRP by centrifugation at 400 × g for 10 min at RT and then resuspended in 100 µl diluent C (Sigma, PKH26 kit), then 2 µl of the diluted dye PKH26 was added, mixed and the cells were incubated for 5 min at RT. After the incubation, 1 ml of Tyrode buffer (pH 7.4) was added, the cells centrifuged at 400 × g for 10 min at RT and resuspended in 3 ml PBS/EDTA/Heparin. Labeling efficiency of the platelets was checked under the microscope and using FACS analysis (data not shown).

### Infusion of platelets into wildtype mice

Freshly isolated PKH26 labeled platelets from 1 mouse (3–5 × 10^7^) were slowely infused transcardially into anesthetized 6-months old C57BL6 wildtype mice using a 21 gauge syringe. Briefly, mice were anesthetized, the heart exposed, the needle passed through the cut left ventricle into the ascending aorta, the heart/needle clamped, an incision was made into the right atrium and then 3 ml of the PKH26 labeled platelets were slowely infused. The brain was immediately taken and directly sectioned into 110 µm thick organotypic brain slices using a vibratome. These vibratome slices were immediately checked under the microscope to identify PKH26 labeled platelets in the slices and only slices with >10 platelets per section were used. Slices were then carefully placed onto 0.4 μm membrane inserts (Millipore PICM03050) on a 6-well plate. The organotypic brain slices were cultured at 37 °C and 5% CO_2_ with 1.2 ml/well culture medium for 2 weeks as described by us in detail elsewhere^26^. After culturing, slices were fixed for 3 hr with 4% PAF and stored in PBS until use.

### Effects of MMP inhibitor

The MMP inhibitor I (CAS 1177749-58-4, Calbochem) was used to test any interaction with MMPs. The MMP inhibitor was dissolved in DMSO and is a cell-permeable potent and reversible MMP-9 inhibitor with an IC_50_ of 5 nM. At high concentrations it also inhibits MMP-2 (IC_50_ = 1.05 µM) and MMP-13 (IC_50_ = 113 nM). In our experiments it was used at a concentration of 1 µM. In order to test any effects of the inhibitor, the inhibitor was added during the infusion as well as during incubation of the slices.

### FACS analysis

FACS analysis was performed as reported by us and others previously^23,48^. Briefly, platelets were isolated, dissolved in tyrode buffer and 50 µl diluted cells were incubated in FACS buffer (2 mM EDTA, 0.5% FCS, in PBS) in BD FACS tubes with 5 µl of the following antibodies: LeoF2 Emfret M025-1 FITC labeled (GPIIbIIIa), XiaB4 Emfret M051-1 FITC labeled (GPIX), Xia X2 Emfret M043-1 FITC labeled (GPIba) and JON/A Emfret M023-2 (GPIIbIIIa activated, PE labeled). The respective IgG controls served as a control. After incubation for 30 min at 4 °C, 1 ml of FACs buffer was added, vortexed, and the cells centrifuged at 300 g for 10 min, then the pellet resuspended in FACS flow and immediately analyzed in a FACScan. In order to stimulate platelets with thrombin, isolated platelets were incubated with 1 mM CaCl2 and 500 µg thrombin (from bovine plasma, Merck 12374) for 30 min in FACS buffer with the respective antibodies and then processed and analyzed the same way.

### Immunostainings

Organotypic brain slices cultured for 14 days were processed for immunofluorescence stainings as described in detail previously^23^. Briefly, brain sections were washed with PBS and incubated in PBS/0.1% Triton (T-PBS) for 30 min at 20 °C while shaking. After incubation, the sections were blocked in T-PBS/20% horse serum (Gibco Invitrogen) 0.2% BSA (SERVA) for 30 min at 20 °C while shaking. Following blocking, brain sections were incubated with primary antibodies against collagen IV (1:500, Abcam ab6586), CD41 (1:2000, abcam ab63323), CD61 (1:200, Thermo Scientific MA1-80862), Aβ (1:1000, Covance SIG-39300, clone 6E10), Iba-1 (1:500, Wako nr. 019-19741), TNFα (1:250, abcam ab34674), MMP-9 (1:250, R&D Systems AF909), MMP-2 (1:250, abcam ab37150) in T-PBS/0.2% bovine serum albumin (BSA) for 2–3 days at 4 °C. The sections where then washed and incubated with the fluoresecent secondary goat (MMP-9), mouse (CE41, CD61, Aβ), rabbit (collagen IV) Alexa-488 or Alexa-647 (1:400, Invitrogen-Life tech, Vienna, Austria) antibody in T-PBS/0.2% BSA for 1 hour at 20 °C while shaking. Finally the sections were washed in PBS, then mounted onto glass slides and coverslipped with Mowiol^®^ 4–88 (Roth, Austria). Alternatively, sections were stained with Thiazine Red or Resorufin (1.6 μg/ml, Sigma, overnight). Vessels in slices were also stained with Lectin-649 (1 hr 1:100, Vector DL-1178) insteadt of collagen-IV. Vessel damage was visualized using Alexa-488 labelled anti-mouse IgG (3 hr 1:400, Invitrogen A11029).

### Analysis

Fluorescence analysis was performed with a Fluorescence microscope Olympus Bx61. Alexa-488 (collagen IV vessels) was visualized in the green channel (EX 480/40 nm, EM 527/30 nm) and PKH26 (red platelets) in the red channel (EX 535/50, EM 610/75). Fluorescence images were aquired using the openlab software (4.0.4).

Confocal microscopy was performed using an SP5 confocal microscope (Leica Microsystems, Wetzlar, Germany) with an HCX PL APO _63 and/or 1.3 NA glycerol objective as reported previously^23^. Three confocal images were analyzed per brain and the volume (µm^3^) of the vessel and platelets were measured with the image software Imaris 8.2, as well as the platelets penetration from the vessel (in µm). Confocal imaging was performed with an argon laser line (set power to 20%) for AlexaFluor 488 (collagen IV vessels, co-stainings for MMP-2, MMP-9, anti-mouse-IgG, Iba-1, TNFα), a DPSS561 nm laser for AlexaFluor 546 (red PKH26 platelets) and HeNE 633 for Alexa 647 (Lectin-649 vessels, co-stainings for CD61, CD41, Aβ). Emission of each fluorophore was detected from 493–556 nm (AlexaFluor 488), 566–628 nm (AlexaFluor 546) and 638–750 nm (Alexa 647). For the control panel the smart gain was set to 250 Volt (V) per turn, smart offset to 0.1 or 1%, zoom to medium, X position to fine, Y position to fine and the resolution was set to 12 bit, pixels size between 40 and 60 pixels, speed to approximately 1000 Hertz (Hz), frame resolution to 1024 × 1024 and the line average between 1–3. General parameters for the sampling intervals were set to X (nm) 60.125, Y (nm) 60.125, Z (nm) 125.885. For the objective correction the Photomultiplier (PMT) was activated and set to a gain of 500–600 V and the Scan Mode from XYZ to XZY. Afterwards “AOBS” was clicked and the settings changed to “Reflection”. The PMT detector ranges were set to min 487 nm and max 556 nm, the bright line was adjusted to the middle of the image on the right monitor using the z-motor of the joystick control and the line was made as bright and thin as possible. Afterwards the session was adjusted to “scan mode again to XYZ” and Reflection unchecked. For our experiment “between lines” in the scanning method was used. For the Deconvolution with the Huygens software the following parameters were used: numerical aperture (1.3), Objective quality (good), coverslip position (µm): Estimate position; Imaging directions: upward, Lens immersion: Glycerine (1.474), Embedding: Glyc.90% (Mowiol) 1.458, Backprojecting pinhole (nm): 307.09; Excitation fill factor: 2.00. The signal/noise per channel was set to 15,15,15, max iterations to 100, the search for background to auto, the background per channel to 0.0, 0.0., 0.0 and the bleaching correction to if possible, brick mode to auto, the quality change threshold (%) to 0.1 the iteration mode to optimized and the padding mode to automatic. After the Deconvolution the images were processed with the Imaris 8.1 sofware for 3D imaging.

To measure the Overlap Volume (µm^3^) a surface was created from channel 1 or from channel 2 with the sofware Imaris 8.2. Then a corresponding channel was selected to mask the surface, the voxels outside the surface were set to 0.000. A new masked channel (e.g. the overlap of channel 2 with the surface 1) was then created. Finally a new surface was created from this new masked channel (representing the volume overlap) to obtain the volume in statistics tab.

### Release assay and ELISAs

The release of MMPs was analyzed in isolated platelets and vessels from wildtype and transgenic mice. Vessels were isolated using a BSA centrifugation step as described in detail by us elsewhere^49^. Briefly, isolated platelets or vessels were resuspended in 100 µl Tyrode buffer and incubated for 60 min at 37 °C; then cells were centrifuged 1900 g 10 min and the supernatent analyzed by ELISA and the pellet measured for total protein using Bradford assay. Values are given as released MMPs per mg protein per 60 minutes. ELISAs were performed as given in the manufactures instructions; mouse MMP-2 and MMP-9 were analyzed using ELISA kits from FineTest (MMP-2: EM0142 and MMP-9: EM0144, Wuhan, China). Briefly, supernatants were added to wells, incubated for 90 min 37 °C, washed, incubated with biotinylated antibodies for 60 min 37 °C, washed, incubated with ABC working solution for 30 min 37 °C, washed and developed using TMB substrate and measured at 450 nm with a Zenyth ELISA reader. Values were correlated to standard curves.

### Statistics

Statistical analysis was performed by One way ANOVA with a subsequent Fisher LSD posthoc test where p < 0.05 was considered as significant. A students T-test was used where 2 groups were compared.

## Electronic supplementary material

Supplementary Figure 1
