# Supplementary material for: Platelets isolated from an Alzheimer mouse damage healthy cortical vessels and cause inflammation in an organotypic ex vivo brain slice model
Source: Sci Rep. 2018 Oct 19;8:15483. doi: 10.1038/s41598-018-33768-2 (PMC6195547; doi:10.1038/s41598-018-33768-2)
Supplement: Supplementary file 1 — Supplementary Figure 1 [file 41598_2018_33768_MOESM1_ESM.pptx]

## Slide 1
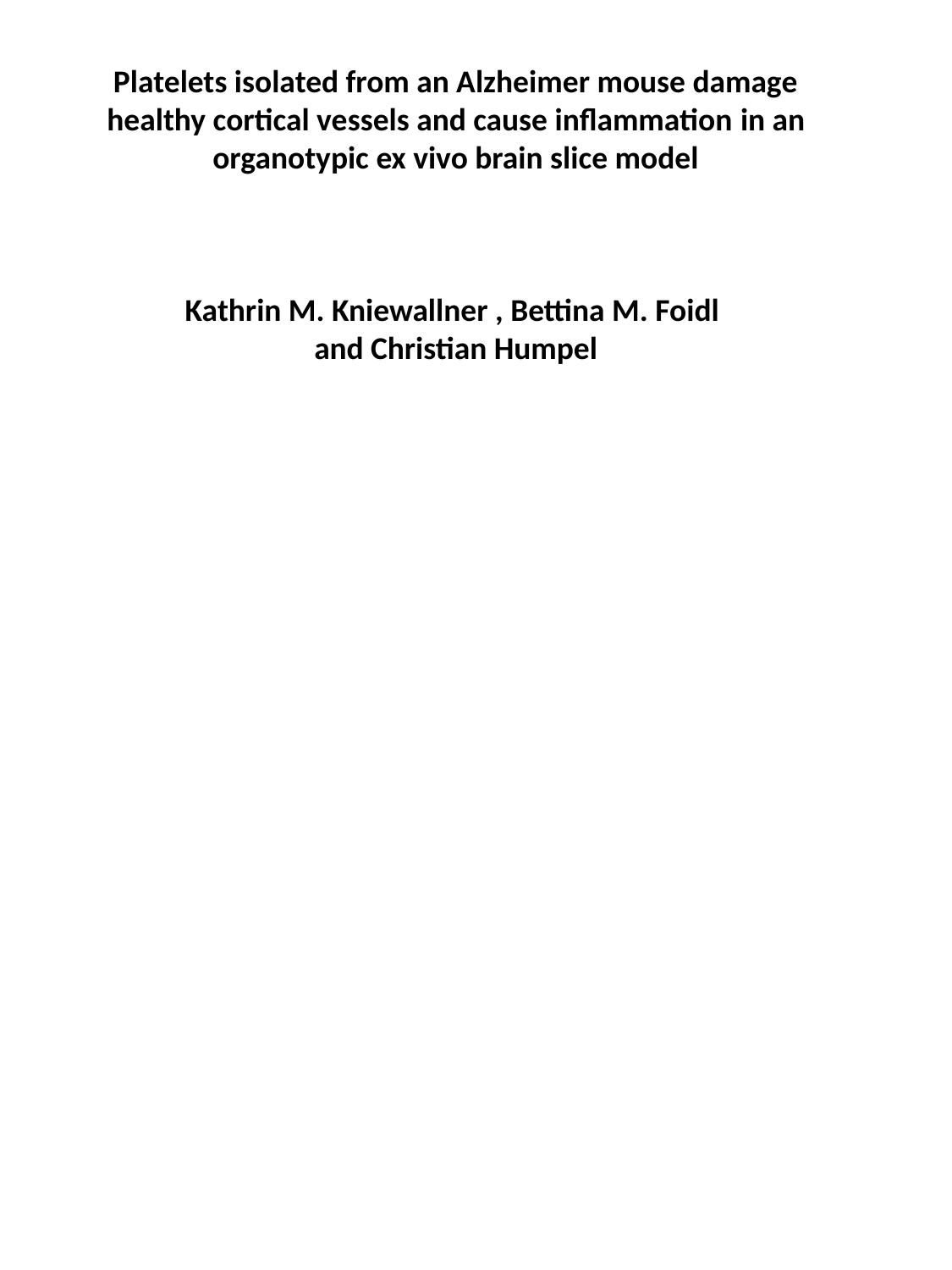

Platelets isolated from an Alzheimer mouse damage healthy cortical vessels and cause inflammation in an organotypic ex vivo brain slice model
Kathrin M. Kniewallner , Bettina M. Foidl
 and Christian Humpel

## Slide 2
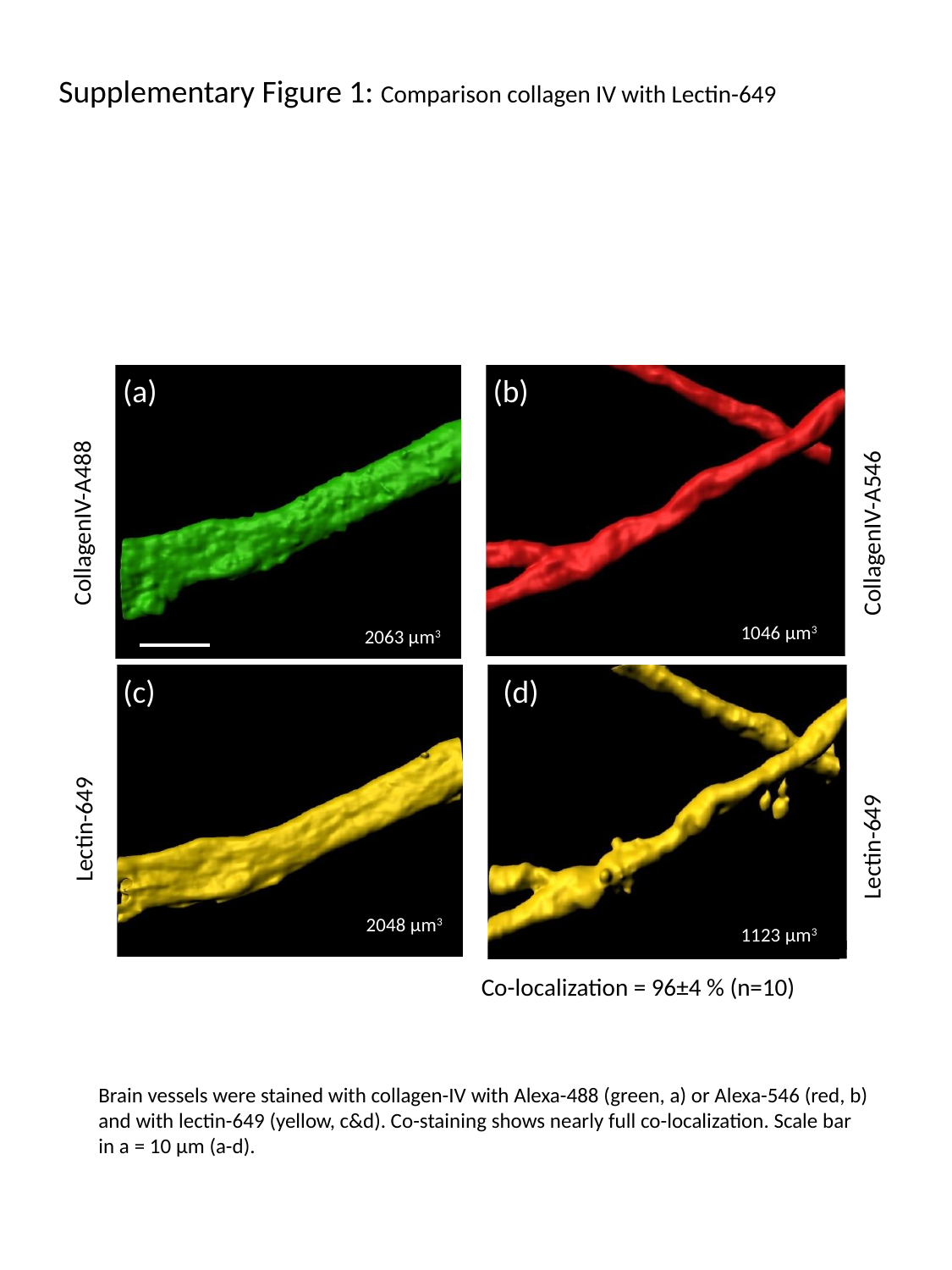

Supplementary Figure 1: Comparison collagen IV with Lectin-649
(a)
(b)
CollagenIV-A488
CollagenIV-A546
1046 µm3
2063 µm3
(c)
1123 µm3
(d)
Lectin-649
Lectin-649
2048 µm3
Co-localization = 96±4 % (n=10)
Brain vessels were stained with collagen-IV with Alexa-488 (green, a) or Alexa-546 (red, b) and with lectin-649 (yellow, c&d). Co-staining shows nearly full co-localization. Scale bar in a = 10 µm (a-d).
